# Efficacy and cost-effectiveness of a physiotherapy program for chronic rotator cuff pathology: A protocol for a randomised, double-blind, placebo-controlled trial

**DOI:** 10.1186/1471-2474-8-86

**Published:** 2007-08-31

**Authors:** Kim Bennell, Sally Coburn, Elin Wee, Sally Green, Anthony Harris, Andrew Forbes, Rachelle Buchbinder

**Affiliations:** 1 Centre for Health, Exercise & Sports Medicine, School of Physiotherapy, University of Melbourne, Australia; 2 Monash Institute of Health Services Research, Monash University, Melbourne, Australia; 3 Centre for Health Economics, Monash University, Melbourne, Australia; 4 Department of Epidemiology and Preventive Medicine, Monash University, Melbourne, Australia; 5 Monash Department of Clinical Epidemiology at Cabrini Hospital, Melbourne, Australia

## Abstract

**Background:**

Chronic rotator cuff pathology (CRCP) is a common shoulder condition causing pain and disability. Physiotherapy is often the first line of management for CRCP yet there is little conclusive evidence to support or refute its effectiveness and no formal evaluation of its cost-effectiveness.

**Methods/Design:**

This randomised, double-blind, placebo-controlled trial will involve 200 participants with CRCP recruited from medical practices, outpatient departments and the community via print and radio media. Participants will be randomly allocated to a physiotherapy or placebo group using concealed allocation stratified by treating physiotherapist. Both groups will receive 10 sessions of individual standardised treatment over 10 weeks from one of 10 project physiotherapists. For the following 12 weeks, the physiotherapy group will continue a home exercise program and the placebo group will receive no treatment. The physiotherapy program will comprise shoulder joint and spinal mobilisation, soft tissue massage, postural taping, and home exercises for scapular control, posture and rotator cuff strengthening. The placebo group will receive inactive ultrasound and gentle application of an inert gel over the shoulder region. Blinded assessment will be conducted at baseline and at 10 weeks and 22 weeks after randomisation. The primary outcome measures are self reported questionnaires including the shoulder pain and disability index (SPADI), average pain on an 11-point numeric rating scale and participant perceived global rating of change. Secondary measures include Medical Outcomes Study 36-item short form (SF-36), Assessment of Quality of Life index, numeric rating scales for shoulder pain and stiffness, participant perceived rating of change for pain, strength and stiffness, and manual muscle testing for shoulder strength using a handheld dynamometer. To evaluate cost-effectiveness, participants will record the use of all health-related treatments in a log-book returned to the assessor monthly. To test the effect of the intervention using an intention-to-treat analysis, linear regression modelling will be applied adjusting for baseline outcome values and other demographic characteristics. Participant measures of perceived change will be compared between groups by calculating the relative risks and their 95% confidence intervals at each time point using log binomial regression.

**Discussion:**

Results from this trial will contribute to the evidence regarding the effectiveness of a physiotherapy program for the management of CRCP.

**Trial registration:**

NIH Clinical Trials Registry # NCT00415441

## Background

Shoulder disorders are a common cause of musculoskeletal morbidity in the community [1,2], affecting 15–30% of adults at any one time [3] and having their peak prevalence in the mid-to-older age groups [4]. Since moving the shoulder allows placement of the hand, compromised shoulder function impacts substantially on tasks essential to daily living such as dressing, eating, personal hygiene and work [5]. Shoulder pain often impairs the ability to sleep, thus affecting mood and concentration. Hence shoulder disorders can lead to considerable disability, reductions in health-related quality of life, absenteeism from work and substantial utilization of health care resources [5-7].

Shoulder disorders are a common reason for seeking medical care – in Australia, they account for 1.2% of all general practice encounters, being third only to back (3.8%) and neck complaints as musculoskeletal reasons for primary care consultations [8]. They also account for up to 10% of all referrals to physiotherapists [9]. Shoulder disorders are often recalcitrant with persisting pain and disability from 12 [10] to 18 months [11] in up to 50% of cases. Chronic shoulder conditions may require surgical intervention in 15–28% of individuals [12]. Thus, they are a relevant health problem for clinicians, funding providers and health-care policy makers.

Although there is controversy as to exact definitions for different shoulder diagnostic categories, a large proportion of shoulder problems can be classified under the term 'chronic rotator cuff pathology' (CRCP). The term (or its variants such as impingement syndrome) includes a spectrum of pathologies (tears, inflammation, tendonitis, degeneration) involving contractile and other local structures around the shoulder joint [13,14] giving rise to similar signs and symptoms. Pain is the main feature, particularly on overhead movement, at night, and when lying on the affected side. Examination may reveal a painful arc during shoulder elevation, pain on resisted shoulder abduction, external rotation or internal rotation, and a positive impingement test (ref). Restricted shoulder range may be present but is mostly related to pain rather than stiffness per se [15]. Unlike other shoulder conditions, such as adhesive capsulitis, global shoulder passive restriction is not a feature. Diagnosis is traditionally based on history and clinical findings. Investigative procedures such as x-ray, ultrasound or magnetic resonance imaging may be used to demonstrate CRCP or to exclude other less common shoulder pathologies.

It is thought that shoulder impingement is involved in the initiation and/or perpetuation of CRCP. This occurs when the greater tuberosity impinges against the coracoacromial arch in shoulder elevation causing mechanical irritation of local structures [15]. While there is speculation as to whether the pathology seen with CRCP is primary or secondary to impingement, a number of factors can play a role in CRCP [15,16]. In particular, if the rotator cuff muscles do not function in a co-ordinated manner with each other as well as with the principal muscles that move the humerus and scapula, then inadequate stabilisation of the humeral head may occur during shoulder elevation [17,18]. Poor shoulder, cervical and thoracic posture as well as tight structures can also contribute to dysfunctional movement patterns [19,20] and ultimately chronic pathology and pain.

Physiotherapy is often the first line of management for CRCP [21]. In a survey of approximately 800 primary care medical practitioners, 79% stated that their usual treatment of a patient with CRCP would be referral to a physiotherapist [21]. Physiotherapy aims to reduce pain and disability by improving the biomechanics and movement patterns of the shoulder complex rather than by treating the pathology *per se*. In clinical practice, patients with CRCP receive a combination of physiotherapy interventions in order to effectively address the modifiable factors contributing to pain and dysfunction.

While physiotherapy is commonly prescribed for CRCP, at present there is little conclusive evidence to support or refute its effectiveness, and no formal evaluation of its cost-effectiveness. In a Cochrane review, 11 trials were identified that evaluated physiotherapy for CRCP [22]. No firm conclusions were able to be drawn given the variable methodological quality of the trials and the fact that many tested a single physiotherapy modality with few testing combined modalities despite this being the most common way in which shoulder disorders are treated in practice. Furthermore, eight of the 11 (72%) CRCP trials evaluated electrotherapy modalities which are generally not recommended by expert clinicians as the most appropriate physiotherapy modality to treat CRCP. From the trials in CRCP or in mixed shoulder disorders that were included in the review, there was some evidence to propose that exercise may be effective, with additional benefit from mobilisation [23-26]. Recent trials identified since this Cochrane review still fail to clarify the effectiveness of physiotherapy for CRCP given differences in the treatment program, comparison group and outcomes [27-30]. The need for further clinical trials in this area is supported by the conclusions and recommendations of other recent systematic reviews [31-33].

Therefore, the aim of this trial is to determine whether a 10-week multimodal physiotherapy program reflective of current physiotherapy practice improves pain, disability and health-related quality of life and is more cost-effective than placebo in individuals with CRCP.

## Methods/Design

### Design

**Figure 1:**
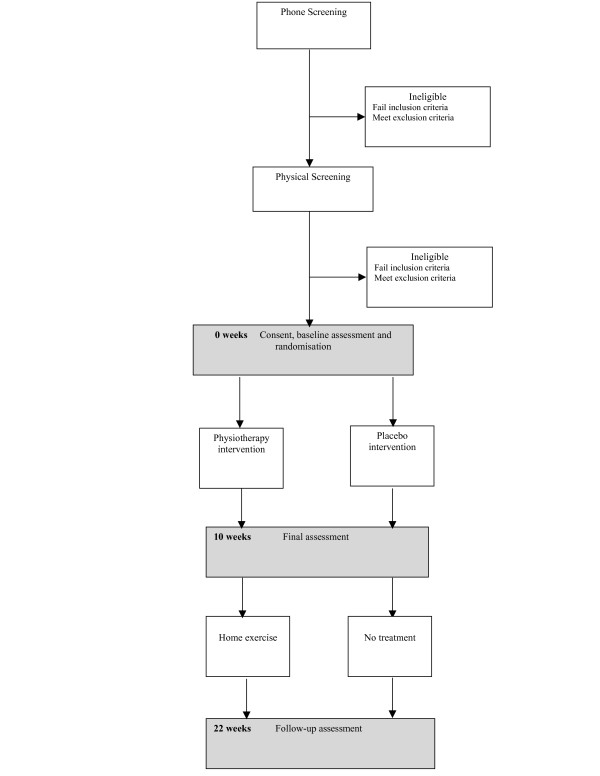
Trial profile.

This will be a randomised placebo controlled trial with blinded participants and outcome assessors (Figure 1). The trial comprises a 10-week intervention and a 12-week follow-up (Figure 1). Participants will be assessed immediately before treatment (baseline), immediately after treatment (final) and at 22 weeks (follow-up) post randomisation. The assessments will be performed by the same assessor who will be blind to group allocation.

### Participants

A community sample will be recruited in Melbourne, Australia through orthopaedic and rheumatology outpatient clinics, community-based practices and general practitioners as well as by advertising in print and radio media. Inclusion criteria will be: (i) aged over 18 years; (ii) shoulder pain for greater than 3 months; (iii) pain severity on movement rated at least 4/10 on an 11 point numeric rating scale; (iv) pain on active abduction or external rotation and; (v) positive shoulder impingement quick test [34]. Exclusion criteria will be: (i) resting shoulder pain of greater than 7/10 in severity; (ii) reason to suspect a complete rotator cuff tear eg. substantial shoulder weakness, a positive drop-arm sign or a high riding humerus observed on plain xray; (iii) prior shoulder surgery; (iv) radiological evidence of shoulder osteoarthritis, shoulder joint calcification or prior fracture; (v) systemic pathology including inflammatory joint disease or neoplastic disorders; (vi) more than 50% restriction of passive range of motion in two or more planes; (vii) shoulder pain referred from vertebral structures diagnosed via spinal clearing tests [35]; (viii) symptoms of complex regional pain syndrome; (iix) active intervention in last 3 months including corticosteroid/hydrodilatation injection or physiotherapy; (ix) anti-inflammatory medication in past two weeks; (x) inability to understand written and spoken English.

A diagnosis of CRCP by a medical doctor supported by a plain x-ray of the shoulder will be necessary for potential inclusion. All volunteers will then be required to undergo a physical examination with a physiotherapist to screen for signs and symptoms, and to exclude shoulder pain that is not due to rotator cuff origin (eg, neural or cervical referral, adhesive capsulitis). Participants with bilateral CRCP symptoms will be asked to indicate which shoulder is the most symptomatic. This shoulder will be treated. Those with equally symptomatic shoulders will receive treatment for the shoulder of the dominant limb.

The Royal Melbourne Hospital Human Research Ethics Committee approved the study (Project #2001.115) and all participants will provide written informed consent.

### Randomisation and allocation

Following a physical screening examination, volunteers who meet the inclusion criteria will be accepted as participants and undergo baseline assessment at the University of Melbourne. Following baseline assessment, participants will be stratified by treatment site and randomly assigned in permuted block of six and eight to either physiotherapy or placebo treatment groups. The randomisation sequence will be generated using a computer-generated table of random numbers by the study biostatistician (AF). Allocations will be sealed in opaque and consecutively numbered envelopes kept in a locked location. These will be opened in sequence by an independent administrator not involved in eligibility assessment, outcome assessment or treatment. Allocation will be revealed to the treating physiotherapist by fax before the participant presents for treatment.

### Interventions

Ten musculoskeletal physiotherapists located around metropolitan Melbourne will implement both interventions. All treatments will be individual sessions lasting 30–45 minutes, twice weekly for the first 2 weeks, once a week for the next 4 weeks, then once a fortnight in the last 4 weeks (a total of 10 treatments). Participants will be requested to refrain from seeking other forms of treatment during the trial. However, due to ethical considerations, analgesia will be permitted. Use of medications and other therapies will be recorded in a logbook.

The treatments will be standardized and the therapists will be trained to deliver both treatments prior to the study. A comprehensive treatment manual will be produced and the importance of following the protocol emphasised. For the physiotherapy treatment, it is considered important to allow the therapists to adjust the intensity of the standardised treatment techniques to match the participants' capabilities. Thus the number of repetitions of the exercises can be reduced. Deletion of techniques will be permitted if participants find them too painful. At the end of each treatment session for each participant, the physiotherapist will complete a checklist to ensure compliance with the standard approach.

All participants will be requested not to seek other forms of treatment during the study but use of analgesia will be permitted and recorded in a log-book.

#### Physiotherapy intervention group

A standardized treatment protocol has been devised (SC) based on the literature and on the results of a formal written survey we conducted involving 10 musculoskeletal physiotherapists around Australia, considered by the profession to be experts in treating shoulder conditions. These physiotherapists were asked to indicate which of a series of interventions they would generally use at three stages of a 10-week program when managing a typical patient with CRCP. The results showed that the most commonly employed modalities were scapular retraining (100%), rotator cuff exercises (100%), spinal (83%) and shoulder joint mobilisation (50%), soft tissue massage (66%) and taping (50%). Less emphasis is placed on electrotherapy modalities (33%) with few using these, particularly past the first three weeks of treatment (Coburn et al unpublished data).

The aims of the resultant physiotherapy intervention are to i) decrease pain; ii) improve functional range of shoulder motion; iii) improve scapular control; iv) strengthen scapular stabilisers and rotator cuff muscles; v) improve posture and thoracic extension range of motion; vi) regain normal shoulder biomechanics. The treatment has five components comprising soft tissue massage, passive mobilisation of the glenohumeral joint, scapula retraining, spinal mobilisation, and home exercises (Table 1). Cognitive behavioural strategies will also be incorporated including education, goal-setting, motivation and positive reinforcement.

**Table 1:** Components of the physiotherapy intervention

**Treatment component**	**Dosage**
Soft tissue massage	6 mins each position
Anterior and posterior shoulder tissues performed in supine and sidelying respectively	
Glenohumeral joint mobilisation	4 × 30 seconds each position
Anteroposterior and inferior joint glides in supine with shoulder at 45° and 90° abduction respectively	
Thoracic spine mobilisation (T1-8)	Grade IV on each level – 4 mins in total
Performed in prone using a central posteroanterior technique	
Cervical spine mobilisation (C5-7)	Grade IV on each level – 4 mins in total
Performed in prone using unilateral posteroanterior technique on both sides	
Scapular retraining	Weeks 1 and 2 only
In sidelying, therapist passively moves shoulder through range from elevation/protraction to retraction/depression then assisted by participant then independently. Isometric holds in retraction/depression	15 reps × 5 reps with 10 sec holds
Postural taping	Continuous (day and night) for two weeks
Taping of the shoulders and scapula to encourage scapular retraction and depression and thoracic extension	Re-applied after one week by the therapist
Exercises	Home program:
Supervised and performed as home program	Twice daily in first two weeks
	Once a day thereafter

##### Soft tissue massage

Deep massage of the soft tissue structures around the shoulder joint will be performed for six minutes in two positions. The posterior joint capsule and scapular shoulder musculature will be massaged in sidelying while the anterior shoulder structures including the supraspinatus, long head of biceps and pectoralis minor tendons will be massaged in supine.

##### Glenohumeral joint mobilisation

Anteroposterior and inferior glenohumeral joint mobilisations will be performed with the participant in supine and with the shoulder in 45 degrees abduction and 90 degrees abduction respectively [35]. Grade IV oscillations (into 50% resistance) will be maintained for 30 seconds and repeated four times.

##### Scapular retraining

The aim of scapular retraining is to improve control of scapular movement, particularly to avoid excessive elevation and protraction, so as to optimise the functional position of the shoulder. The therapist will passively guide the participant from a neutral shoulder girdle posture to an adducted, depressed position, then to an upwardly rotated, elevated position. The participant will then perform the movement actively for several repetitions followed by a 10 second hold in the adducted, depressed posture. This will be repeated 5 times. Postural taping will also be worn full time for the first two weeks. The therapist will firstly apply a protective skin barrier followed by non-rigid, hypoallergenic tape to provide skin protection, and then rigid strapping tape for postural adjustments. The shoulder taping technique aims to encourage a retracted, depressed scapular posture and thoracic extension.

##### Spinal joint mobilisations

Mobilisation techniques will be performed to improve range of cervical and thoracic motion, particularly thoracic extension. They will include unilateral lower cervical spine (C5-7) and central upper-mid thoracic spine (T1-8) posteroanterior mobilisation techniques with the participant in prone lying [35]. The therapist will use a Grade IV force at each level, on both sides for the cervical spine and centrally for the thoracic spine. The duration of this treatment will be four minutes for each technique.

##### Home exercises

These are predominantly designed to increase rotator cuff and scapular muscle strength (Table 2). Most exercises require the participant to incorporate their scapular retraining with strengthening of the rotator cuff muscles. Some exercises reinforce and facilitate correct posture. These exercises will be taught and performed during each treatment session and exercise progressions will be introduced regularly throughout the course of the treatment program. Resistance for specific exercises will be provided by hand weights or elastic theraband. Participants will be asked to perform the exercises daily, except during the first week of treatment where exercises will be completed twice per day. Compliance will be monitored via a weekly log book completed by the participant. During the follow-up, the physiotherapy group will be requested to continue daily home exercises.

**Table 2:** Description of specific exercises

**Name**	**Description**	**Dosage**	**Weeks performed**
Scapular setting	Sitting, isometric hold of scapula in retracted and depressed position	5 sec hold × 5 reps	Week 1 then maintained in all exercises
Self-resisted isometric ER	Standing sideways to wall. Upper arm squeezing a towel roll against body, elbow bent with forearm pushing into wall	5 sec hold × 5 reps	Weeks 1&2
Active ER	Sitting with shoulder in 45° Abd resting elbow and forearm on table in IR. Taking shoulder into ER	10 reps × 2	Week 1&2
Shoulder shrugs	Standing with arms slightly abducted and actively elevating scapula then lowering slowly	10 reps × 2 handweight	Week 1&2
Pectoralis minor stretch	Supine with arms in 45° Abd and elbows bent to 90°. Shoulders rotate into ER to stretch muscle	5 reps with 10 sec hold × 2	Weeks 2–6
Wall push up	Standing arms length from wall, hands at shoulder height and shoulders in 45° Abd. Body lowered to wall and then pushed away	5 reps × 2	Weeks 2–10
Chin tuck	Standing with head and back against a wall. Chin tucked in toward neck	5 reps × 10 sec holds	Week 2
Resisted external rotation	Sidelying with affected shoulder uppermost, elbow bent to 90° and holding weight in hand. Weight lifted up toward ceiling with upper arm against body	10 reps × 2 using hand weight	Week 2
Thoracic extension over towel	Supine on a firm surface with arms by side and lying on towel roll placed horizontally on floor at level of maximum thoracic curve	1–3 mins hold	Weeks 3–6
Resisted scapular setting – elbow extension with shoulder neutral	Standing, arm by side and elbow bent holding theraband attached in front at shoulder height. Elbow straightened and slowly flexed whilst keeping scapula in set position	10 reps × 2 using theraband	Weeks 3–6
Resisted external rotation	Standing, elbow bent to 90° and forearm along stomach and holding onto theraband at waist height. Keeping elbow in to side, pulling against theraband to perform ER	10 reps × 2 using theraband	Weeks 3&4
Resisted internal rotation	Standing, elbow bent to 90° and shoulder in ER and holding onto theraband at waist height. Keeping elbow in by side, pulling against theraband to perform IR	10 reps × 2 using theraband	Weeks 3&4
Resisted horizontal row	Standing, both arms outstretched holding onto theraband attached at waist height. Both arms pulled back toward trunk with elbows flexed	10 reps × 2 using theraband	Weeks 3–10
Resisted external rotation in supported 90° Abd	Sitting with shoulder supported in 90° Abd on table and forearm resting on table holding a weight in hand. Weight lifted toward ceiling keeping elbow on table	10 reps × 2 using hand weight	Weeks 5&6
Resisted internal rotation in supported 90° Abd	Sitting with shoulder supported in 90° Abd on table and forearm resting on table holding theraband attached behind. Hand taken to table to perform IR	10 reps × 2 using theraband	Weeks 5&6
Corner stretch	Standing with one hand on each corner wall at shoulder height and elbows bent. Leaning in toward corner to stretch anterior shoulder and thoracic spine	5 reps × 10 sec holds	Weeks 5–10
Resisted scapular setting – elbow flexion	Standing with hands at chest height, elbows bent and holding onto theraband which is then stretched apart by trying to straighten both elbows	10 reps × 2	Weeks 7–10
Resisted external rotation in unsupported Abd	ER performed in standing with shoulder unsupported in 45° scapular plane, elbow bent and holding theraband attached in front	10 reps × 2	Weeks 7–10
Resisted internal rotation in unsupported Abd	IR performed in standing with shoulder unsupported in 45° scapular plane, elbow bent and holding theraband attached behind	10 reps × 2	Weeks 7–10

ER = external rotation; IR = internal rotation; Abd = abduction

#### Placebo group

As for many procedural interventions, it is difficult to design a placebo treatment that fully mimics a physiotherapy program. However, our aim is to control for the effect of regular contact with a therapist, the belief that treatment will assist CRCP and the therapeutic environment. Participants in the placebo group will receive the same number and length of visits as those in the physiotherapy group but will receive only sham ultrasound and light application of a non-therapeutic gel. Participants in the placebo group will receive no instruction in exercise techniques and no manual therapy. We have used an identical placebo protocol in completed clinical trials of physiotherapy for patellofemoral pain syndrome [36], knee osteoarthritis [37] and shoulder adhesive capsulitis [38]. In these trials, between 68% to 83% of participants in the placebo group thought they had received 'real' physiotherapy or were unsure. Blinding index was 0.49 (bootstrap 95% CI 0.40 to 0.56), interpreted as moderate success of blinding in one study [38]. The placebo participants will not receive any intervention or complete any home exercises during the 12 week follow-up period.

### Blinding

To maintain blinding, the plain language statement and consent procedures will inform participants that they have an equal chance of receiving real or placebo physiotherapy but will not disclose details of the actual treatments. A blinded examiner will perform all outcome assessments. Participant blinding will be optimised by using a realistic placebo intervention and by ensuring participants do not attend for treatments or assessments concurrently. Participants will also be requested to refrain from discussing their treatment with the outcome assessor. At trial completion, participants will nominate which group they believed they have been allocated to. The data manager and statistician will be unaware of treatment allocation until completion of analyses.

### Outcome assessment

Demographic information will be collected including age, sex, duration of symptoms, previous investigations and treatment, history of medical conditions, and medication use. Expectation of a beneficial treatment effect will be scored on an ordinal scale from 1 to 5 with higher scores indicating higher expectations.

A number of outcome measures will be collected for this study (Table 3). A combined shoulder pain and disability index (SPADI) will be used to measure changes in shoulder pain and function at final and follow-up time points. This is a self-administered, shoulder-specific index consisting of 13 items divided into two subscales: pain (five items) and disability (eight items) [39]. Responses to each item are recorded on a 11 point Likert scale where 0 = "no pain" or "no difficulty" and 10 = "worst imaginable pain" or "so difficult it required help" for the pain and disability items respectively. The SPADI score is calculated by summing then averaging the two subscales to give a score out of 100 (higher score more pain/disability). It has acceptable test-retest reliability, construct validity and responsiveness [39-41]. We have confirmed the clinimetric properties of this index and found it to be more responsive than other shoulder-specific questionnaires (effect sizes 1.01–1.69) (Buchbinder et al unpublished data).

**Table 3:** Outcome measures

**Primary Outcomes**	**Measurement**
Shoulder pain and disability index (SPADI)	13 items scored on an 11 point Likert scale
Average pain over past week	11 point horizontal numeric rating scale (end descriptors of 0 = no pain and 10 = worst pain possible)
Participant perceived global rating of change overall	Ordinal scale (1-much worse, 2-slightly worse, 3-no change, 4-slightly better, 5-much better)

**Secondary Outcomes**	

Worst pain, and pain on 3 self selected activities in past week	11 point horizontal numeric rating scale (end descriptors of 0 = no pain and 10 = worst pain possible)
Amount of stiffness, weakness, and interference to activities of daily living in past week	11 point horizontal numeric rating scale
Participant perceived global rating of change in pain, strength, and stiffness	Ordinal scale (1-much worse, 2-slightly worse, 3-no change, 4-slightly better, 5-much better)
Health-related quality of life	• SF-36 • Assessment of Quality of Life index (AQol)
Isometric muscle strength of shoulder abduction, internal rotation and external rotation	Hand held dynamometer

**Other measures**	

Compliance	• Number of therapy visits • Physiotherapy group: Completion of home exercises via log-book
Adverse effects	Log-book and open probe questionning

Average pain, worst pain and pain on three self-selected activities (over the past week) will be measured by separate 11-point numeric rating scales [12] numbered in 1 cm intervals. The amount of weakness, stiffness and interference with activities of daily living (over the past week) will be measured similarly.

Participant perceived global rating of change overall and in pain, strength, and stiffness (since commencement) will be recorded on separate 5 point Likert scales (1-much worse, 2-slightly worse, 3-no change, 4-slightly better, 5-much better). Measuring participant perceived improvement using a rating of change scale has been shown to be a clinically relevant and stable concept for interpreting truly meaningful improvements from the individual perspective [42]. A successful outcome will be defined a priori as 'much better' on the rating scale.

Health-related quality of life will be measured using the Medical Outcomes Study 36-item short form (SF-36) (8 subscales scaled from 0–100 where a higher score represents better health) [43]. This the most widely used generic measure of health related quality of life and permits comparison of the impact of disease and treatment across studies and populations. The clinimetric properties of the SF-36 have been well established on samples from diverse populations [43,44]. We will also use the Assessment of Quality of Life (AQoL) instrument. The AQoL comprises 15 items on ordinal scales with four levels per item covering five dimensions (illness, independent living, social relationships, physical senses and psychological wellbeing). It produces a single utility index that ranges from -0.04 (worst possible health-related quality of life) to 1.00 (full health-related quality of life). The AQoL has strong psychometric properties [45,46]. The AQoL can also be converted into a utility index to calculate quality adjusted life years (QALY).

Isometric shoulder strength for shoulder abduction, internal and external rotation will be measured using the Nicholas Manual Muscle tester (Lafayette, USA). For shoulder abduction, participants will be positioned in supine with the shoulder in 90 degrees of abduction and the elbow flexed to 90 degrees. The dynamometer will be positioned on the lateral surface of the distal humerus, proximal to the lateral epicondyle. Measurements of external and internal rotation will be performed in sitting with the arm by the side against a folded towel, the elbow flexed to 90 degrees and the forearm in midprone. The dynamometer will be positioned on the distal forearm. One warm up trial followed by three maximal contractions will be performed and the median reading taken. We previously measured 12 people with CRCP on two occasions three days apart. Intraclass correlation coefficient (ICC 2,3) values were 0.95 for abduction, 0.96 for internal rotation and 0.93 for external rotation indicating that test retest reliability was excellent.

A number of other measures will be obtained (Table 3). Participant compliance will be obtained by recording the number of physiotherapy sessions attended (out of a maximum number of 10). Those in the physiotherapy group will complete a daily log-book to record the number of home exercise sessions completed. Adverse events and the use of co-intervention will be recorded in a log-book and by open-probe questioning by the assessor at trial completion. Log-books will be posted back to the assessor on a monthly basis and checked for completion. At the final and followup measurement time points, study participants will be asked to indicate which treatment they believe they have received and reasons for that choice to assess the success of blinding.

Information on direct health care costs, direct non-health care costs and production losses over the 22 weeks will be collected by a logbook posted back to the assessor on a monthly basis and checked for completion. Direct health care costs will include costs of physiotherapy attendance (assumed zero in the placebo group), additional health provider visits, tests, prescription and over the counter medication, professional home care and hospitalisation. These will be valued using published prices for medical costs. Direct non-health care resources will include use of paid and unpaid help, lost time and travel, and number of lost days at work.

### Sample size

Sample size was calculated based upon the ability to detect a 10-point difference in improvement in SPADI score, previously reported to indicate a clinically important improvement (or worsening) of shoulder function [41]. Applying power calculations appropriate for analysis of covariance (adjusting for baseline SPADI score), to detect a difference in 10 week SPADI of 10 units assuming a common between-participant standard deviation of 27 and a baseline to 10 week correlation in SPADI scores of 0.45 (from our pilot study), 91 participants per group will be required to achieve 80% power at a two-sided 5% significance level. Including the 10 week and further 22 week follow-ups in a repeated measures analysis increases the power to 85% assuming a conservative correlation of 0.8 between all post-baseline measurements and a uniform physiotherapy effect. We will allow for a 10% loss to follow-up and aim to recruit 100 participants per group.

### Data analysis

All analyses will be conducted on an intention-to-treat principle using all randomized participants. Missing data will be replaced by the last score carried forward. Demographic characteristics and baseline data will be summarised by descriptive statistics. An index will be computed to assess the success of blinding [47]. This index takes the value one for complete blinding and zero for complete lack of blinding.

For outcomes measured using an essentially continuous scale, differences in mean change from baseline to each time point will be compared between groups using linear regression modelling adjusting for baseline levels of the outcome measure. Model assumptions will be checked by standard diagnostic plots [48]. For analysis across all time points simultaneously, accounting for repeated measurements, we will estimate the differences between groups using generalised estimating equation models for the post-baseline measurements with adjustment for baseline measurements, a robust variance and unstructured working correlation [49]. Constancy of the difference between groups over time will be assessed by fitting models which include a term for the interaction between treatment and time. Sensitivity analyses include repetition of analyses with calculation of bootstrap standard errors, and identification of influential individuals by sequentially omitting each participant and refitting the model.

Participant measures of perceived improvement following physiotherapy or placebo treatments will be compared by calculating the relative risks and their 95% confidence intervals at each time point using log binomial regression [50]. Repeated measures relative risk calculations will be performed using generalised estimating equations with a logarithmic link function, robust variance and unstructured correlation [49]. As above, models including a term for the interaction between treatment and time will be fit to assess the constancy of the difference between groups over time.

The primary economic evaluation will take the form of a cost effectiveness study with a range of outcome measures including the incremental cost per extra person with a clinically significant improvement in pain, per extra person perceived to be recovered, and per extra quality adjusted life years (using the AQoL over 22 weeks). A social perspective on costs will be taken that includes resource use incurred both by health services and by the participant irrespective of the source of payment. The inclusion of time/productivity gains is controversial and the cost effectiveness ratios will be calculated with and without these indirect costs. All health care costs will be included, however to reduce the impact of extreme values, if inpatient hospital costs are unrelated to CRCP they will be excluded. Standard methods of economic evaluation alongside a clinical trial [51] will be used to evaluate the differences in resource use and health outcomes over 12 months between groups. The statistical analysis of costs data will be similar to outcome data although adjustments for overdispersion may be necessary. Confidence intervals for incremental cost effectiveness will be calculated directly using non-parametric bootstrapping [52]. In addition we will calculate a cost effectiveness acceptability curve based for a range of hypothetical money values of outcomes [53]. This will be done using individual cost and outcome data over the 22 weeks or, if adjustments for imbalance at baseline are necessary, using regression analysis [54]. Hypothetical money values will be taken from the decision making literature but the trial will also ask patients in each arm of the trial their willingness to pay for the treatment prior to and after treatment. This will not only provide money values for the calculation of net benefits but also provide evidence on the influence of health experience on the value of health outcome to patients.

## Discussion

This study uses a double-blind randomised controlled trial design to investigate whether a multimodal physiotherapy program has greater effects on pain, disability and health-related quality of life and is more cost-effective than placebo in people with CRCP. The benefits of physiotherapy for this patient group have not been well established in the literature and there is no information on its cost effectiveness.

At present there is no gold standard diagnostic test for CRCP. Our criteria for inclusion into the study are based on clinical assessment performed by a medical doctor and a physiotherapist together with a shoulder xray to assist in differential diagnosis. While imaging techniques such as magnetic resonance imaging or ultrasound may further improve diagnostic accuracy particularly if performed by a single experienced operator [55], these still lack sensitivity for certain pathologic features [56,57] and are costly. We wished to reflect the population that present to primary care for assessment and treatment and who in many cases will not have these investigative procedures performed.

Physiotherapy generally encompasses a multimodal approach to treatment. Therapists utilise a variety of techniques such as mobilisation, soft tissue massage, taping, exercise prescription and education. As there is no 'gold standard' physiotherapy for CRCP, we chose to use a standardised program rather than employ a pragmatic design whereby therapists chose the program based on their own clinical reasoning and experience. This was done to ensure a consistent approach and to allow replication of the program tested. A number of treating therapists were included to increase the external validity of the results.

We chose a placebo treatment as the comparator rather than no treatment as we wished to evaluate the specific effects of physiotherapy over and above those gained from regular contact with a caring therapist. There is some debate in the literature about whether it is appropriate to use a placebo treatment for interventions such as physiotherapy or acupuncture where it is difficult to isolate the direct and indirect effects of the therapy [58]. It has been argued that these effects are unlikely to be distinct, divisible and additive and that using a placebo controlled trial design will not detect the whole treatment effect and may generate false negative results. However, we believe that the primary goal of physiotherapy is to improve the modifiable impairments associated with CRCP using various techniques and as such it should provide greater benefits than those simply due to the therapeutic environment. The project physiotherapists will treat an equal proportion of experimental and placebo group participants to ensure that any effects of the personality of each therapist and the treatment environment are equally distributed.

It is anticipated that all participants will be recruited by the middle of 2007 with data acquisition completed six months later. The results from this trial will contribute to evidence based recommendations for the effectiveness of a physiotherapy program in the management of CRCP.

## Competing interests

The author(s) declare that they have no competing interests.

## Authors' contributions

KB, SC and RB conceived and designed the trial protocol. KB, RB, SG, SC, AH and AF procured the project funding. SC and SG designed the physiotherapy intervention. AH designed the economic analysis. AF designed the statistical analysis. EW is the research assistant and blinded assessor. KB drafted the manuscript and RB, EW, SC, SG, AH and AF contributed to the manuscript. All authors read and approved the final manuscript.

## Pre-publication history

The pre-publication history for this paper can be accessed here:


